# Isolation and characterization of *Listeria* species from rodents in natural environments in China

**DOI:** 10.1038/emi.2017.28

**Published:** 2017-06-07

**Authors:** Yan Wang, Liang Lu, Ruiting Lan, Joelle K Salazar, Jingli Liu, Jianguo Xu, Changyun Ye

**Affiliations:** 1 State Key Laboratory of Infectious Disease Prevention and Control, National Institute for Communicable Disease Control and Prevention, Collaborative Innovation Center for Diagnosis and Treatment of Infectious Diseases, Chinese Center for Disease Control and Prevention, Beijing 102206, China; 2 School of Biotechnology and Biomolecular Sciences, University of New South Wales, Sydney, NSW 2052, Australia; 3 Department of Biology, Illinois Institute of Technology, Chicago, IL, USA

**Keywords:** antimicrobial resistance, *Listeria*, molecular characteristic, natural environments, rodents

## Abstract

*Listeria* is ubiquitous in a variety of environments and can be isolated from a wide range of animal hosts. Rodents are capable of carrying pathogenic bacteria in their intestines, such as *Listeria*, and can disseminate those pathogens into the natural environment and to where human activity occurs. In this study, we investigated the occurrence and antimicrobial susceptibility of *Listeria* spp. isolated from wild rodents found in natural environments in China. We collected 341 intestinal fecal samples of rodents from five different regions of China, all representing different rodent habitats. The antimicrobial susceptibility of the *Listeria* spp. isolates obtained were firstly assessed using the Kirby–Bauer disk diffusion method. Thirty-one samples were positive for *Listeria* spp., of which 11 were positive for *Listeria monocytogenes* and seven were positive for *Listeria ivanovii*. Other species identified include *Listeria innocua*, *Listeria fleischmannii* and *Listeria floridensis*. All *Listeria* spp. isolates were sensitive to the majority of the antimicrobials tested, but largely resistant to oxacillin (94.1%) and cefuroxime (70.6%). All *L. monocytogenes* isolates were further characterized by serotyping, multi-locus sequence typing (MLST) and pulsed-field gel electrophoresis (PFGE). *L. monocytogenes* strains were grouped into three serotypes, five sequence types and five pulsotypes (PTs) by serotyping, MLST and PFGE, respectively. Almost half of the isolates (five of 11) belonged to serotype 1/2b, ST87 and PT1. This study determined that *Listeria* is carried in the intestinal tracts of wild rodents from multiple regions at a low rate, filling an epidemiological data gap on *Listeria* in natural environments in China.

## INTRODUCTION

Until recently the genus *Listeria* was thought to consist of six species, including *L. monocytogenes*, *L. innocua*, *L. welshimeri*, *L. ivanovii*, *L. seeligeri* and *L. grayi.* However, another 11 novel species have been reported since 2009: *L. marthii*, *L. fleischmannii*, *L. floridensis*, *L. rocourtiae*, *L. weihenstephanensis*, *L. cornellensis*, *L. aquatica*, *L. riparia*, *L. grandensis*, *L. booriae* and *L. newyorkensis*.^1, 2, 3, 4, 5, 6, 7^ Only two species, *L. monocytogenes* and *L. ivanovii*, are pathogenic due to their respective species-specific virulence determinants. *L. monocytogenes* can cause severe invasive infections in both humans and animals, while *L. ivanovii* rarely causes infections in humans but is an important cause of infection in other animals, particularly in ruminants.^8, 9^ Human infections by other *Listeria* species, such as *L. seeligeri* and *L. innocua*, are rare and are seen mainly in immunocompromised individuals.^10, 11^ Human listeriosis, which is mainly caused by the consumption of *L. monocytogenes* in contaminated food products, has low morbidity but high mortality rates. The symptoms of listeriosis in humans are non-specific, varying from mild to severe illness. Immunocompetent adults may suffer a self-limiting febrile gastroenteritis.^12^ In contrast, immunocompromised individuals, including people with severe underlying disease conditions, the elderly and newborns, may suffer septicemia and central nervous system infections.^13^ Pregnant women may suffer miscarriage, preterm delivery or stillbirth, although only flu-like symptoms are manifested.^14^

*Listeria* has been shown to be ubiquitously distributed in a variety of environments due to its adaptability. For example, *Listeria* can survive at a broad range of pH (4.5–9.2), temperature (0–45 °C) and salt concentrations (up to 10% NaCl).^15^ Most *Listeria* spp. isolates are susceptible to many antimicrobials, except for some modern cephalosporins, dalfopristin/quinupristin, pipemidic acid, oxacillin and aztreonam.^16^ The occurrence and antimicrobial resistance of *Listeria* from various food products and food-processing environments have been well studied.^17, 18^ Recently, *Listeria* spp. isolated from food products or food-processing environments which are resistant to multiple antimicrobial agents have been reported worldwide.^19, 20^ However, there is a lack of published information on the occurrence and antimicrobial resistance of *Listeria* spp. in wild animals from natural environments. In these environments, rodents could represent a reservoir for many pathogens. Shedding of *Listeria* spp., especially *L. monocytogenes* and *L. ivanovii*, in the feces of rodents could contaminate food products or food-processing environments by direct or indirect transmission paths. The present study was undertaken to determine the occurrence and antimicrobial resistance of *Listeria* spp. in rodents from natural environments.

*L. monocytogenes* isolates are classified into four lineages by a large number of subtyping methods.^21, 22, 23^ The majority of *L. monocytogenes* isolates belong to lineage I (including serotype 1/2b, 3b, 4b, 4d and 4e) or lineage II (including 1/2a, 3a, 1/2c and 3c). A number of studies have shown that the majority of human listeriosis cases were associated with lineage I isolates, while major *L. monocytogenes* contaminations of food were associated with lineage II isolates. *L. monocytogenes* isolates assigned to lineage III or lineage IV, including serotype 4a and 4c, are rare and mostly isolated from ruminants.^24^ Pulsed-field gel electrophoresis (PFGE) and multi-locus sequence typing (MLST) have been widely used for the epidemiological investigation of *L. monocytogenes* and source tracking of specific strains in outbreaks. Further molecular characterization for *L. monocytogenes* isolates from rodents was performed to provide insight on epidemiological features of this foodborne pathogen from natural environments.

## MATERIALS AND METHODS

### Sample collection

According to the medical research regulations of the Ministry of Health China, the present study was approved by the ethics committee of the National Institute for Communicable Disease Control and Prevention, China CDC (Approval NO. ICDC2014003). In this study, 341 intestinal fecal samples of rodents were collected from five regions (Tibet, Hainan, Guangdong, Fujian and Shanxi province) in China during September 2014–June 2015. The sampling areas comprised five different habitat types, including the junction area of farm and woodland, woodland, cassava field, grassland–shrubland and waste-grassland (Table 1). The captured rodents were autopsied, and ~1 g of fecal content from the cecum and colon were collected in 5 mL of Brain Heart Infusion Broth (BHIB) containing 15% glycerol. The samples were stored at 4 °C for ~two weeks prior to experiments.

### Isolation, identification and confirmation of *Listeria* spp.

*Listeria* strains were isolated according to the ISO 11290 method with modifications. The intestinal feces were introduced into 10 mL Half-Fraser broth and incubated at 30 °C for 24 h. Subsequently, 0.5 mL of the primary enrichment cultures were transferred to 4.5 mL Fraser broth and incubated at 37 °C for 48 h. A loopful of secondary enrichment was streaked onto Chromogenic *Listeria* Agar (Oxoid, Basingstoke, UK) and incubated at 37 °C for 24–48 h. After incubation, colonies suspected of being *Listeria* spp. based on color and morphology were selected for identification. Bacterial colonies were identified using 16S rDNA amplification and sequencing and the API *Listeria* test (bioMérieux, Marcyl’Etoile, France). All confirmed *Listeria* isolates were stored in BHIB containing 15% glycerol at −80 °C.

### Antimicrobial susceptibility testing

Sensitivity of the *Listeria* isolates to 16 antimicrobials (10 μg gentamicin, 30 μg kanamycin, 10 μg streptomycin, 10 units penicillin G, 10 μg ampicillin, 1 μg oxacillin, 30 μg chloramphenicol, 5 μg rifampicin, 10 μg imipenem, 30 μg vancomycin, 2 μg clindamycin, 15 μg erythromycin, 30 μg tetracycline, 1.25/23.75 μg trimethoprim/sulfamethoxazole, 5 μg ciprofloxacin and 30 μg cefuroxime) was assessed using the disk diffusion technique according to the Clinical and Laboratory Standard Institute (CLSI). Briefly, pure frozen culture was transferred to BHIB and incubated at 37 °C overnight. A cell suspension was prepared by suspending colonies in 0.85% NaCl (w/v) until the turbidity was equal to 0.5 MacFarland standards; the suspension was spread onto the surface of Mueller–Hinton agar (Oxoid). The diameter of the inhibition zone surrounding each disk was measured after 18–24 h of incubation at 37 °C. The results for each antimicrobial were classified as sensitive, intermediate or resistant according to the CLSI criteria for *Staphylococci* spp.^25^ *Streptococcus* *pneumoniae* ATCC 49619 was used as a control strain.

### Serotyping, MLST and PFGE

*L. monocytogenes* serotype was identified by a combination of multiplex PCR and traditional slide agglutination. The multiplex PCR was performed by targeting the genes *lmo0737*, *lmo1118*, *ORF2110*, *ORF2819* and *Listeria*-specific *prs* described by Doumith *et al.*^26^ When the serogroups were identified, only the antiserum against somatic antigens was used. MLST based on seven house-keeping genes (*abcZ*, *bglA*, *cat*, *dapE*, *dat*, *ldh* and *lhkA*) was performed according to the method of Ragon *et al.*^27^ The scheme and genotypic data are available at http://bigsdb.web.pasteur.fr/listeria/. Minimum spanning tree analysis was inferred using BioNumerics (Version 5.10, Applied Maths, Belgium). PFGE of the *L. monocytogenes* strains was performed using the primary restriction enzyme *Asc*I according to the standard operating procedure by PulseNet of Centers for Disease Control and Prevention.^28^ Similarities between the digestion profiles of strains were analyzed by unweighted pair group method with arithmetic mean using BioNumerics software (Version 5.10, Applied Maths).

### Statistical analysis

*X*^*2*^ test or Fisher’s exact test, as appropriate, was performed in SAS 9.4 (SAS Institute Inc., Cary, NC, USA) to check the significant effect of the regions and the types of rodents on the occurrence of *Listeria* spp. A *P*-value <0.05 was considered as statistically significant. In order to reduce the sample collection bias, the rodents were categorized into six types based on genera, including *Rattus* (*n*=130), *Niviventer* (*n*=72), *Apodemus* (*n*=67), *Mus* (*n*=33), *Bandicota* (*n*=25), and others (including *Phaiomys* (*n*=9), *Cricetulus* (*n*=3) and *Microtus* (*n*=2)).

## RESULTS

### Occurrence of *Listeria* spp. in feces of rodents

Thirty-one out of 341 fecal samples were found to be positive for *Listeria* spp. (Table 1). Three samples contained two *Listeria* species with two samples containing *L. monocytogenes* and *L. innocua*, and one sample containing *L. monocytogenes* and *L. fleischmannii*. The 34 *Listeria* spp. isolates included 11 *L. monocytogenes* (32.4%), seven *L. ivanovii* (20.6%), 10 *L. innocua* (29.4%), five *L. fleischmannii* (14.7%) and one *L. floridensis* (2.94%). By region and habitat, the prevalence rates of *Listeria* ranged from 5.3% (Guangdong) to 25.8% (Tibet) and from 3.2% (woodland) to 16.7% (grassland–shrubland; Table 2). The incidence of *Listeria* in Tibet was significantly higher than in the other regions (*P*<0.05). The prevalence rates of *Listeria* in six types of rodents varied from 0% to 16% (Table 2), however, the differences were not statistically significant (*P*>0.05).

### Antimicrobial resistance of *Listeria* spp. strains

All the *Listeria* spp. isolates were tested for antimicrobial susceptibility (Table 3). The most frequent antibiotic resistance was to oxacillin (94.1%), followed by cefuroxime (70.6%), clindamycin (20.6%) and tetracycline (11.8%). All isolates were sensitive to gentamicin, kanamycin, penicillin G, ampicillin, imipenem and vancomycin. All of the *L. fleischmannii* isolates were resistant to clindamycin. Moreover, one of the *L. innocua* isolates was found to be resistant to seven antibiotics tested, including streptomycin, oxacillin, chloramphenicol, clindamycin, trimethoprim/sulfamehtoxazole, tetracycline and cefuroxime.

### Genotypic characterization of *L. monocytogenes* isolates

**Figure 1:**
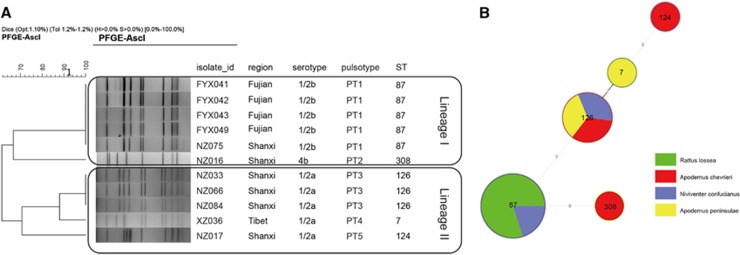
(**A**) Pulsed-field gel electrophoresis-based dendrogram representing *L. monocytogenes* strains isolated from rodents of wild fields in China. The 11 *L. monocytogenes* isolates were divided into two lineages by serotyping, MLST and PFGE. The corresponding data, including the name of the isolate (isolate_id), region, serotype, pulsotype and ST are shown. (**B**) The minimum spanning tree of the five sequence types of *L. monocytogenes* isolates from rodents in wild environments in China. The corresponding sequence type and isolated region are displayed within the circles. The size of each circle corresponds to the isolate count, and the color within the circles represents the species of the rodents. multi-locus sequence typing, MLST; pulsed-field gel electrophoresis, PFGE; sequence type, ST.

The 11 *L. monocytogenes* isolates were typed by serotyping, MLST and PFGE. Five isolates were serotype 1/2a, five were 1/2b and one was 4b. By MLST, the 11 isolates were divided into five sequence types (STs). There were five ST87, three ST126 and one each of ST7, ST124 and ST308. Isolates with the same ST also shared the same pulsotype, for example, all ST87 isolates were PT1, while all ST126 isolates were PT3 (Figure 1A). Four of the five ST87/PT1 strains were isolated from waste-grassland in Fujian. The minimum spanning tree of the *L. monocytogenes* isolates determined that there was no correlation between STs of *L. monocytogenes* and the species of rodents, although the sample set was small (Figure 1B).

## DISCUSSION

Although there have been many reports of contamination of food products and food-processing environments with *L. monocytogenes* and other *Listeria* spp., there have been limited studies on the occurrence and characteristics of *Listeria* spp. in wild animals from natural environments. In this study, we found that the incidence of *Listeria* spp. in the feces of rodents was 9.97%, with *L. monocytogenes* and *L. ivanovii* at 3.23% and 2.05%, respectively. Tibet had the highest incidence of *Listeria* and was statistically significantly different from the other regions (*P*<0.05), although the number of positive samples was small. It is interesting to note that *L. ivanovii* was only isolated from Tibet. The higher isolation rate in the Tibet plateau may be attributed to the activities of a variety of wildlife, particularly ruminants, such as yaks, sheep and deer. However, this is unlikely due to the altitude. Kristina *et al.* found that a higher *L. ivanovii* isolation rate occurred in wildlife reserve regions and sites near the habitats of wild and domestic ruminants; the altitudes of the study sites were <500 m, which are much lower than the Tibet plateau (above 3000 m).^29^

The prevalence of *L. monocytogenes* in rodents from natural environments was lower than those reported in different food products in China (ranging 5.3%–20%).^30, 31, 32^ Higher prevalence in the latter may be because food products and food-processing environments provide a very suitable environment for the proliferation of *Listeria* spp. However, a recent survey on various food products from three large cities in central China observed a lower prevalence of *L. monocytogenes* (2.3%).^33^

There has of yet been no reports focusing on *Listeria* spp. prevalence in wild animals in China. For *Listeria* spp., higher prevalence levels from rodents were reported in Japan by Iida *et al.* and Inoue *et al.*, with rates of 17.1% and 24.5%, respectively.^34, 35^ In addition, higher isolation rates of *Listeria* spp. were found in wild birds according to the studies of Yoshida *et al.* and Hellstrom *et al.*, with rates of 24.5% and 53%, respectively.^36, 37^ In contrast, lower prevalence rates of *Listeria* spp. were reported in domestic animals (with the exception of pig at 12.2%), including cats (0%), chickens (4.7%), dogs (2%), and cattle (5.1%) by Iida *et al.*,^34^ as well as in wild non-rodent mammals (6.1%) by Yoshida *et al.*^36^ For *L. monocytogenes*, higher isolation rates have been reported in rodents (6.5% and 5.7%)^34, 35^ and wild birds (36%) in Japan,^37^ as well as in livestock (5.9%) and wild animals (3.7%) in Canada.^38^ In contrast, lower isolation rates have occurred in domestic animals ranging from 0% to 1.92% in the study of Iida *et al.*^34^ and in wild non-rodent mammal animals (0.96%) and birds (0.5%) in the report of Yoshida *et al.*^36^ For *L. ivanovii*, the isolation rates reported in rodents (1.6%) and wild non-rodent mammal animals (0.16%) were lower than those reported in this study.^35, 36^ However, few studies have reported the prevalence of this *Listeria* species. Generally, different isolation rates have occurred in different hosts from different regions with no apparent trends. Overall, although the prevalence of *Listeria* spp., especially *L. monocytogenes* and *L. ivanovii*, in wild rodents from natural environments in China was low, surveillance on pathogenic *Listeria* in wild animals from natural environments as a reservoir is important to public health.

Many published studies have determined that the rate of antimicrobial resistance in *Listeria* is relatively low but has been increasing.^39, 40, 41^ Similarly, we determined that almost all of the *Listeria* isolates were susceptible to the antibiotics commonly used to treat human listeriosis. Only one isolate was found to be resistant to trimethoprim/sulfamethoxazole, an alternative drug for patients allergic to penicillin.^42^ Also, all of the *L. fleischmannii* isolates and the one *L. floridensis* isolate were resistant to clindamycin; the vast majority of the isolates were resistant to cefuroxime. Oxacillin resistance has previously been associated with *L. monocytogenes*, *L. innocua* and *L. welshimeri* isolates from various sources, including food products, the environment, animals and humans.^17, 43^ It is interesting to note that the *L. fleischmannii* strains studied in Bertsch *et al.*^3^ harbored a transferable transposon that confers resistance to clindamycin. It would be interesting to determine if our *L. fleischmannii* isolates also carry the resistance transposon. In addition, intermediate resistance against clindamycin was observed in nearly half of *L. monocytogenes* isolates (45%) and most of the *L. innocua* isolates (8, 80%). All but one of the *L. ivanovii* isolates exhibited intermediate resistance to streptomycin. Among the five *Listeria* species in this study, *L. innocua* overall displayed the highest resistance. Notably, one *L. innocua* isolate was resistant to seven of the antibiotics tested. Gomez *et al.* reported that 43.1% and 13.9% *L. innocua* strains isolated from meat products and meat-processing environments were one or two antimicrobials resistance and multidrug resistance, respectively.^43^ High resistance to oxacillin, clindamycin and cefuroxime, as reported previously, may be intrinsic. However, resistance and intermediate resistance to many other antibiotics were surprising as *Listeria* residing in wild rodents would not face selection pressure from antibiotic use. A continuous surveillance of emerging resistance in *Listeria* is important in combating antibiotic resistance in human infections as the resistant isolates from wild animals in natural environments may serve as reservoirs of resistance genes.

In this study, subtyping of *L. monocytogenes* isolates by serotyping, PFGE and MLST provided further insights into the molecular characterization of this pathogen found in rodents in natural environments. All of the 11 *L. monocytogenes* isolates were classified into two previously defined lineages (Figure 1A). Serotype 1/2b and 4b strains belong to lineage I, whereas serotype 1/2a strains belong to lineage II. The serotypes found in this study were all primarily associated with human listeriosis serotypes.^24^ The high prevalence of serotype 1/2a in this study was consistent with the results of other studies from China reporting that serotype 1/2a is one of the predominant serotypes from food products and food-processing environments in China.^19, 44^ Serotypes 4b and 1/2c had also been reported as common serotypes in food products and food-processing environments by different surveys,^19, 45^ although only one 4b and no 1/2c *L. monocytogenes* isolates were identified in this study. By MLST, the predominant ST found was ST87 which was in more than one rodent species and region. ST87 is also the predominant ST which causes human infections.^46^ However the other four STs found in this study were not commonly found in contaminated foods or human infections in China.^45, 46^

*L. monocytogenes* strains were primarily isolated from two of the five regions: Fujian (four strains) and Shanxi (five strains). The *L. monocytogenes* strains displayed different distribution characteristics between these two regions. In Fujian, the *L. monocytogenes* strains which were isolated from *Rattus lossea* in waste-grassland, had identical subtypes, with serotype 1/2b, ST87 and PT1, suggesting that this subtype persisted in this region. In Shanxi, the *L. monocytogenes* strains which were isolated from different species of rodents in the junction area of farm and woodland, were differentiated into four subtypes, demonstrating more genetic diversity. More studies will be required to further assess the genetic diversity of *L. monocytogenes* in these different regions and in different rodent species.

In conclusion, this is the first investigation of the occurrence and characteristics of *Listeria* spp. in wild rodents from natural environments in China. *Listeria* was carried in the intestinal tracts of wild rodents generally at low frequencies from multiple regions in China. However, by strain characteristics, *L. monocytogenes* from wild rodents possesses a potential health risk as some of the same serotypes are most frequently isolated from human infections. Therefore, rodents could spread the pathogen in natural environments by the fecal–oral route, and also to human living environments, potentially leading to disease in humans. *Listeria* spp. which were resistant to antibiotics were isolated in this study. There is a rising threat attributed to the transfer of antimicrobial resistance from non-pathogenic *Listeria* to pathogenic *Listeria* species. This study fills an epidemiological gap on *Listeria* in natural environments in China.

## Figures and Tables

**Table 1:** Combined data on fecal samples of rodents collected and incidence of *Listeria* spp.

**Region**	**Habitat of rodents**	**Species of rodents**	**Number of samples (number of positive sample)**
Tibet	Junction area of farm and woodland	*Apodemus draco*	1 (0)
		*Apodemus peninsulae*	11 (2)
		*Microtus clarkei*	2 (0)
		*Niviventer confucianus*	5 (2)
	Grassland–shrubland	*Cricetulus kamensis*	2 (2)
		*Cricetulus longicaudatus*	1 (0)
		*Phaiomys leucurus*	9 (0)
Hainan	Cassava field	*Niviventer fulvescens*	17 (0)
		*Rattus andamanensis*	17 (0)
		*Rattus lossea*	16 (3)
Guangdong	Waste-grassland	*Rattus norvegicus*	17 (1)
		*Bandicota indica*	1 (0)
		*Mus musculus*	23 (0)
		*Niviventer confucianus*	5 (1)
		*Rattus andamanensis*	2 (0)
		*Rattus lossea*	28 (2)
Fujian	Woodland	*Bandicota indica*	5 (0)
		*Mus musculus*	6 (1)
		*Niviventer fulvescens*	8 (0)
		*Rattus lossea*	11 (0)
		*Rattus norvegicus*	1 (0)
	Waste-grassland	*Bandicota indica*	19 (6)
		*Mus musculus*	4 (0)
		*Niviventer fulvescens*	7 (0)
		*Rattus lossea*	34 (5)
		*Rattus norvegicus*	1 (0)
Shanxi	Junction area of farm and woodland	*Apodemus chevrieri*	35 (4)
		*Apodemus draco*	17 (0)
		*Apodemus peninsulae*	3 (1)
		*Niviventer confucianus*	30 (4)
		*Rattus nitidus*	3 (0)

**Table 2:** Summary data on the occurrence of *Listeria* spp. according to regions, habitat and species of rodents

	**Number of sample**	***L. monocytogenes***	***L. ivanovii***	***L. innocua***	***L. fleischmannii***	***L. floridensis***	**Number** ***Listeria***	**Positive samples (%)**
*Region*								
Tibet	31	1	7	0	0	0	8	25.8
Hainan	50	0	0	0	3	0	3	6.0
Guangdong	76	0	0	2	1	1	4	5.3
Fujian	96	4	0	3	1	0	8	8.3
Shanxi	88	6	0	5	0	0	11	12.5
*Habitat of rodents*								
Waste-grassland	141	4	0	4	2	1	11	7.8
Junction area of farm and woodland	107	7	5	5	0	0	17	15.9
Cassava field	50	0	0	0	3	0	3	6.0
Woodland	31	0	0	1	0	0	1	3.2
Grassland–shrubland	12	0	2	0	0	0	2	16.7
*Species of rodents*								
*Apodemus chevrieri*	35	3	0	2	0	0	5	14.3
*Apodemus draco*	18	0	0	0	0	0	0	0.0
*Apodemus peninsulae*	14	2	3	1	0	0	6	42.9
*Bandicota indica*	25	0	0	0	0	0	0	0.0
*Cricetulus kamensis*	2	0	2	0	0	0	2	100.0
*Cricetulus longicaudatus*	1	0	0	0	0	0	0	0.0
*Microtus clarkei*	2	0	0	0	0	0	0	0.0
*Mus musculus*	33	0	0	1	0	0	1	3.0
*Niviventer confucianus*	40	2	2	2	1	0	7	17.5
*Niviventer fulvescens*	32	0	0	0	0	0	0	0.0
*Phaiomys leucurus*	9	0	0	0	0	0	0	0.0
*Rattus andamanensis*	18	0	0	0	0	0	0	0.0
*Rattus lossea*	89	4	0	2	4	1	11	12.4
*Rattus nitidus*	3	0	0	0	0	0	0	0.0
*Rattus norvegicus*	19	0	0	2	0	0	2	10.5
*Rattus rattus sladeni*	1	0	0	0	0	0	0	0

**Table 3:** Antimicrobial resistance profile of *Listeria* spp. isolates tested in this study

Antimicrobial agent	Disk content (μg)	*L. monocytogenes*			*L. ivanovii*			*L. innocua*			*L. fleischmannii*			*L. floridensis*		
		S	I	R	S	I	R	S	I	R	S	I	R	S	I	R
Gentamcin	10	11	0	0	7	0	0	10	0	0	5	0	0	1	0	0
Kanamycin	30	11	0	0	7	0	0	10	0	0	5	0	0	1	0	0
Streptomycin	10	11	0	0	1	6	0	9	0	1	5	0	0	1	0	0
Penicillin G	10U	11	0	0	7	0	0	10	0	0	5	0	0	1	0	0
Ampicillin	10	11	0	0	7	0	0	10	0	0	5	0	0	1	0	0
Oxacillin	1	0	0	11	1	1	5	0	0	10	0	0	5	0	0	1
Chloramphenicol	30	11	0	0	7	0	0	9	0	1	5	0	0	1	0	0
Rifampin	5	11	0	0	7	0	0	10	0	0	4	1	0	1	0	0
Imipenem	10	11	0	0	7	0	0	10	0	0	5	0	0	1	0	0
Vancomycin	30	11	0	0	7	0	0	10	0	0	5	0	0	1	0	0
Clindamycin	2	6	5	0	7	0	0	1	8	1	0	0	5	0	0	1
Erythromycin	15/30	10	0	1	7	0	0	9	1	0	5	0	0	1	0	0
Trimethoprim-sulfamethoxazole	1.25/23.75	11	0	0	7	0	0	5	0	5	5	0	0	1	0	0
Tetracycline	30	11	0	0	7	0	0	5	0	5	5	0	0	1	0	0
Ciprofloxacin	5	11	0	0	7	0	0	9	1	0	5	0	0	1	0	0
Cefuroxime	30	1	6	4	2	0	5	0	0	10	1	0	4	0	0	1
